# Identification of the novel Np17 oncogene in human leukemia

**DOI:** 10.18632/aging.103808

**Published:** 2020-11-21

**Authors:** Bowen Wu, Yichao Gan, Ying Xu, Zhaoxing Wu, Ganyu Xu, Ping Wang, Chen Wang, Zhipeng Meng, Mengyuan Li, Jiawei Zhang, Haifeng Zhuang, Xuzhao Zhang, Linlin Yang, Jinfan Li, Xiaoxian Gan, Xiaofang Yu, Wendong Huang, Ying Gu, Rongzhen Xu

**Affiliations:** 1 Department of Hematology, Key Laboratory of Cancer Prevention and Intervention, China National Ministry of Education, Key Laboratory of Molecular Biology in Medical Sciences, Zhejiang Province, The Second Affiliated Hospital, College of Medicine, Zhejiang University, Hangzhou 310009, China; 2 Cancer Institute, Zhejiang University, Hangzhou 310009, China; 3 College of Letters and Sciences, University of California-Berkeley, Berkeley, CA 94720, USA; 4 Molecular Oncology Program and Department of Diabetes Complications and Metabolism, Beckman Research Institute, City of Hope National Medical Center, Duarte, CA 91010, USA; 5 Department of Hematology, the First Affiliated Hospital of Zhejiang Chinese Medical University, Hangzhou 310009, China; 6 Department of Pathology, The Second Affiliated Hospital, Zhejiang University School of Medicine, Hangzhou 310009, Zhejiang, China; 7 Zhejiang Academy of Medical Sciences, Hangzhou 310012, China; 8 Institute of Hematology, Zhejiang University, Hangzhou 310009, China

**Keywords:** Np17, leukemia, oncogene, P53, Np9

## Abstract

We previously defined the HERV-K Np9 as a viral oncogene. Here we report the discovery of a novel oncogene, Np17, which is homologous to the viral Np9 gene and predominantly present in Hominoidea. Np17 is located on chromosome 8, consists of 7 exons, and encodes a 16.8kDa nuclear protein with149 amino-acid residue. Functionally, knockdown of Np17 induced growth inhibition of leukemia cells, whereas enforced expression of Np17 promoted growth of leukemia cells in vitro and in vivo. In human leukemia, Np17 was detected in 59.65% (34/57) of acute myeloid leukemia (AML) patients examined and associated with refractory/relapsed AML. Mechanistically, Np17 decreased p53 levels and its mechanism might be involved in recruiting nuclear MDM2 to p53 for ubiquitin-mediated degradation. These findings reveal that Np17 is a novel oncogene associated with refractory/relapsed leukemia.

## INTRODUCTION

Human endogenous retroviruses (HERVs) have resided in the human genome for several million years and makes up 8% of the human genome [1–4]. Although the majority of HERVs are dysfunctional due to multiple nonsense mutations, some are still active and may have potential functions [5–11], especially the HERV type K (HERV-K) family [12–21], which is the most recent entrant into the human genome, having entered 200,000 to 5 million years ago [22], and has been linked to oncogenesis [23]. The viral Np9 transcript, a small regulatory gene of HERV-K type 1, was reported to be exclusively present in tumors and the transformed cells [24]. Interestingly, our previous studies revealed that the viral Np9 protein is an oncoprotein and potently activates a variety of pathways such as β-catenin, ERK, Akt and Notch1, and promotes the growth of human leukemia stem/progenitor cells [1].

Because a number of viral oncogenes have cellular homologs in human cells such as *v-myc* versus *c-myc* [25–30], *v-src* versus *c-src* [31–33], *v-ras* versus *c-ras* [34–36], and *v-abl* versus *c-abl* [37], together with the fact that Np9 is an oncoprotein [1], we hypothesized that there might be undefined cellular homologs of the viral oncogene *np9* in human cells. In this study, we have identified and characterized a novel cellular homolog of HERV-K *np9* gene, which encodes a nuclear oncoprotein of 17kDa associated with human leukemia and is referred to as *np17* gene.

## RESULTS

### Identification of *np17* gene as cellular homolog of the viral *np9* gene

**Figure 1:**
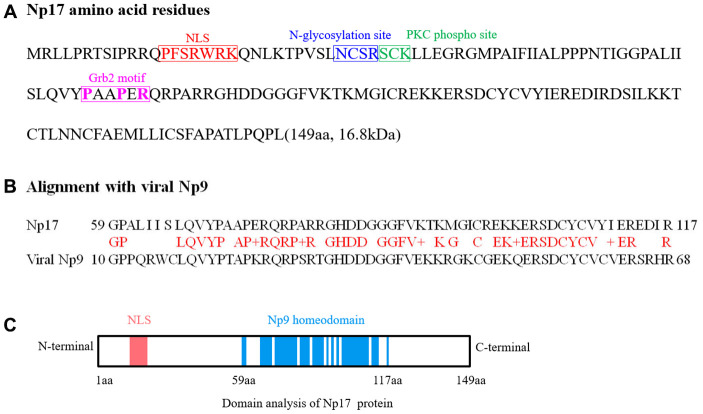
**Analyses of Np17 amino acid sequence and potential motifs.** (**A**) Amino acid sequence of Np17 protein. Putative motifs corresponding to nuclear localization signal (NLS), N-glycosylation site, PKC phospho site and Grb2 motif (P-x-x-P-x-R) are shown in red, blue, green and purple, respectively. (**B**) Alignment of Np17 aa sequence between 59 and 117 aa with viral Np9. (**C**) Schematic representations of putative NLS motif and Np9 homeodomain of Np17 protein.

**Figure 2:**
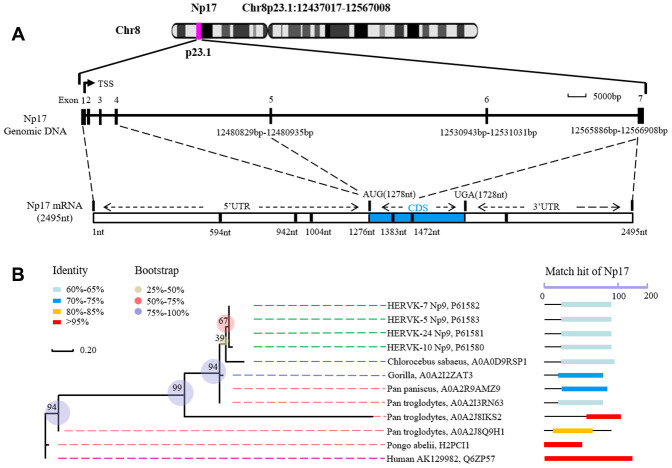
**Structure of human Np17 and evolutionary relationships of Np17 genes.** (**A**) Np17 gene contains 7 exons, in which exons 1-4 are noncoding exons, and exons 5-7 encode Np17 protein. (**B**) Phylogenetic tree based on Np17 protein showing the clustering of Np17 genes in Hominoidea, including Human, Chimpanzee (Pan troglodytes), and Gorilla.

Given that we previously defined the viral *np9* gene, which is aberrantly activated in human leukemia, as a potent oncogene [1], we next attempted to search for cellular homologs of the viral oncogene *np9* in NCBI database by performing alignment using Np9 protein sequence. As expected, we identified a cellular homolog of the viral *np9* gene from Homo sapiens cDNA FLJ26472 fis containing an intact open reading frame (ORF) (450bp) (Supplementary Figure 1). The deduced protein contained 149aa with a predicted molecular mass of ~17kDa (16.8kDa) (Figure 1A). By motif analysis, a nuclear localization signal (NLS) was identified by the PSORT II search program, suggesting a nuclear localization of this putative protein (Figure 1A, 1C). Thus, we referred it to nuclear protein 17 (Np17). A protein kinase C phosphorylation site and an N-glycosylation site were identified by the Gene Runner protein motif research program (Figure 1A, 1C). The identities and positives between Np17 and viral Np9 protein are 67.3% and 76.4% in the Np9 homeodomain region (Figure 1B). To reveal the location and genomic structure of this putative gene, we searched the NCBI database and found that the Np17 gene is located on chromosome 8p23.1 and contains 7 exons spanning ~130kb (Figure 2A). The 1-4 exons are 5’UTR, and the exons 5-7 encode Np17 protein (Figure 2A). We next searched for potential homologs of the Np17 in Uniprot database by performing alignment using human Np17 protein sequence. Unexpectedly, we identified two homologs of the Np17 protein, which are present in Chimpanzee (Uniprot No: H2R9W2) and Gorrila (Uniprot No: G3RQD9), respectively, but not in the database of other species such as rodents. Alignment analysis showed that homologies of human Np17 vs Chimpanzee and Gorrila homologs were 97% and 71%, respectively (Supplementary Figure 2A, 2B). Interestingly, a phylogenetic tree based on Np17 protein showed that *np17* genes were predominantly present in Hominoidea, such as Human, Chimpanzee and Gorilla) (Figure 2B). These results are consistent with the fact that Np9 is restricted to human, chimpanzee and Gorrila [38], suggesting that Np17 might be unique in Hominoidea.

To characterize this putative gene, we amplified the full-length transcript open reading frame (ORF) from mRNAs of both human leukemia cell lines and normal individuals using PCR, and subsequently carried out cloning and sequence analysis. Total 42 cDNA clones were sequenced and contained intact ORF (Supplementary Figure 3). Alignment analysis showed that amino-acid sequences deduced from 42 cDNA clones were highly similar (Supplementary Figure 3), suggesting that Np17 proteins are conserved in human genomes. In addition, we also identified a Grb2 SH3-typical motif (PxxPxR) of the Np17 protein. This motif was conserved throughout all Np17 proteins examined, indicating that a potential role in Np17-mediated disease (Figure 1A).

### *np17* gene encodes a nuclear protein with 17 kDa

**Figure 3:**
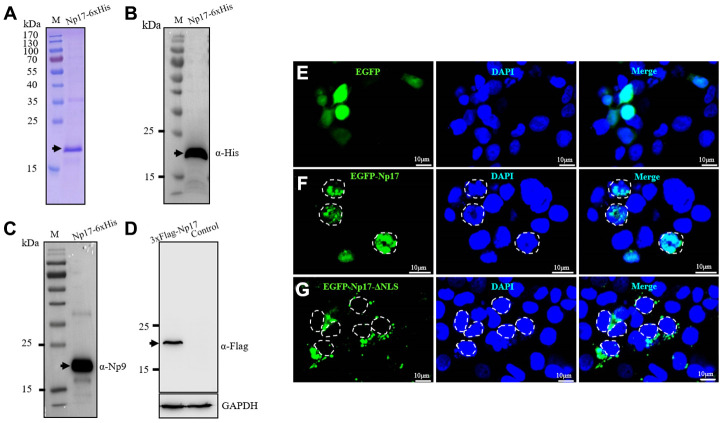
**Np17 gene expresses a nuclear protein of ~17 kDa.** (**A**) Np17 gene expressed a ~17 kDa protein in bacteria. (**B**, **C**) Western blotting result of recombinant Np17 protein with His-tag antibody (**B**) and with Np9 antibody (**C**). (**D**) Np17 gene expressed a ~20 kDa protein in human leukemia cells. (**E**) EGFP alone stained the cell uniformly in HEK293 cells. (**F**) EGFP-Np17 protein is localized predominantly in the cell nucleus in HEK293 cells. (**G**) EGFP-Np17-ΔNLS was localized in the cytoplasm in HEK293 cells.

To determine whether the putative *np17* gene could express protein, we next performed bacterial expression of intact recombinant protein of N*p17* with His tag using Pet-28a construct and observed a protein of ~17 kDa (Figure 3A), which was confirmed by Western blot with His-antibody(α-His) (Figure 3B) and Np9-antibody (α-Np9) (Figure 3C). We then generated a 3xFlag-Np17 plasmid and overexpressed the Np17 protein in human THP-1 leukemia cells. Western blot analysis showed an expression of a protein of ~20 kDa in cell extracts from THP-1-Np17 (Figure 3D). We then investigated the subcellular localization of Np17 protein. A hybrid protein consisting of full-length Np17 and the EGFP fused to the NH2 terminus of Np17 was used in transient transfections of human HEK293 cells. We observed that EGFP alone stained the cell uniformly (Figure 3E). In contrast, EGFP-Np17 proteins were located predominantly in the nucleus (Figure 3F).

To further confirm these observations, we generated a plasmid of EGFP-Np17 mutant with NLS deletion (PFSRWRK): EGFP-Np17-ΔNLS and examined its subcellular distribution under Confocal Laser Scanning Microscope. In contrast to the wild-type Np17 proteins that were predominantly present in the nucleus (Figure 3F), we found that EGFP-Np17-ΔNLS proteins were exclusively present in the cytosol but not in the nucleus (Figure 3G). These results indicate that Np17 is a nuclear protein with NLS domain indeed.

### Detection of native Np17 protein and mRNA in clinical specimens

**Figure 4:**
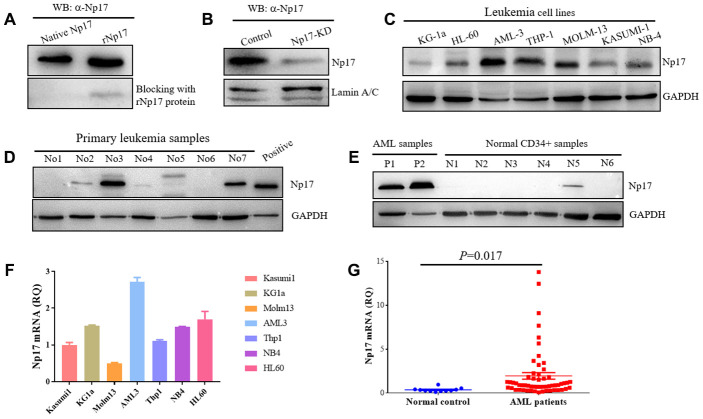
**Human leukemia cells express native Np17 protein and mRNA.** (**A**) Western blot detection of native Np17 protein from a primary leukemia sample with Np17 antibody (α-Np17) and was blocked with recombinant Np17-6xHis protein (rNp17 protein). (**B**) Knock-down of Np17 with shRNA against Np17 significantly reduced nuclear Np17 protein level in THP-1 leukemia cells. (**C**) Western blot detection of native Np17 protein in human leukemia cell lines. (**D**) Western blot detection of native Np17 protein in primary human leukemia samples, the recombined Np17 protein of 293T cells was set as positive control. (**E**) Native Np17 protein was also detected in normal hematopoietic stem cells (CD34+ samples) but its level was lower as compared with AML samples (P1, P2). (**F**, **G**) detection of Np17 mRNA in human leukemia cell lines (**F**) and AML patient samples (**G**) with qRT-PCR.

To confirm whether human cells express native Np17 protein, we raised rabbit polyclonal antibodies against Np17 using the intact recombinant protein of Np17 bacterially expressed from the Pet-28a construct, and detected native Np17 protein in a panel of leukemia cell lines as well as primary leukemia cell samples and normal hematopoietic stem cells by Western blot. The Np17 antibody dilutions for Western blot were 1:1000-5000 and then was confirmed by blocking assay using recombinant Np17 protein, in which a pre-incubation of recombinant Np17 protein (rNp17) potently blocked the binding of Np17 antibody to both native Np17 protein from a primary leukemia cell sample and recombinant Np17 protein (Figure 4A). To further confirm these results, we performed knockdown of Np17 (Np17-KD) with shRNA against Np17. We observed that knockdown of Np17 also significantly reduced nuclear Np17 protein level in THP-1 leukemia cells (Figure 4B). Importantly, we found that native Np17 protein was detected in leukemia cell lines examined (Figure 4C), and primary leukemia samples (No2, No3, No7) (Figure 4D, Supplementary Table 1). However, we found that the Np17 protein levels were not well correlated with its mRNA levels (Figure 4C, 4F), suggesting the potential post-translational modification. In addition, we also found that Np17 proteins in size were not identical in some samples, suggesting modification of Np17 protein with the potential glycosylation or phosphorylation (Figure 1A). Moreover, we found that Np17 protein level was low or absent in normal hematopoietic stem cells (CD34+ cells) (Figure 4E). These results indicate that native Np17 protein is expressed in human leukemia cells indeed (Figure 4B–4D). Consistent with these results, we found that the Np17 mRNA was detected by qRT-PCR in leukemia cell lines (Figure 4F).

### Np17 is aberrantly activated in human acute myeloid leukemia

To address whether Np17 was dysregulated in human leukemia, Np17 mRNA expression was analyzed from 57 patients with newly diagnosed acute myeloid leukemia (AML), which is a rapidly progressing, often fatal hematopoietic malignancy and is the most common leukemia in adults AML(39), and 11 healthy PB samples as a control using qRT-PCR. We observed that the levels of Np17 mRNAs were significantly higher in primary AML patients than those in normal controls (median quantification of mRNA expression after normalization: 1.9580±0.37446 versus 0.3956±0.4330, *P*<0.001) (Figure 4G, Supplementary Table 2), and 59.65% of AML patients (34/57) exhibited higher Np17 mRNAs as compared with normal control. To gain insight into the clinical importance of our findings, we then analyzed Np17 mRNA levels in relation with relapsed/refractory AML (R/R AML). We found that out of 57 AML patients, 22.81% (13/57) of patients exhibited high levels of Np17 mRNA (≥ median RQ value1.9580 of AML patient group), in which the R/R AML rate was up to 84.61% (11/13), whereas the R/R AML rate in AML patients with low levels of Np17 mRNA (<median RQ value1.9580 of AML patient group) was 37.83%(14/37). The R/R AML in Np17-high patients was significantly higher than those in Np17-low AML patients. These data indicate that Np17 expression is highly activated in 22.81% of leukemia patients and high levels of Np17 might be a high risk factor for AML patients.

### Np17 is essential for survival and growth of leukemia cells

**Figure 5:**
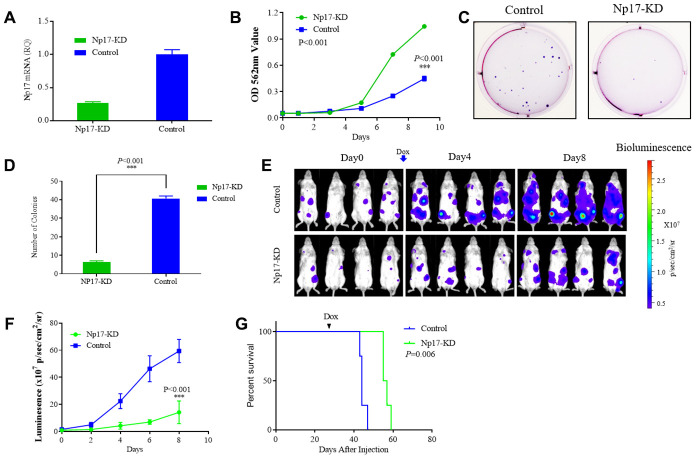
**Np17 is critically required for proliferation and viability of AML Cells.** (**A**) qRT-PCR analysis of Np17 levels in THP-1 cells after DOX-induced Np17-KD. (**B**) Comparison of proliferation curves of THP-1 cells after DOX-induced Np17-KD with control. (**C**, **D**) Representative images and quantification of colony numbers in THP-1 cells after DOX-induced Np17-KD. (**E**–**G**) Leukemia growth inhibition by in vivo Np17 shRNA in an orthotopic mouse model: (**E**) Bioluminescent images of representative mice for each group (n=4). (**F**) Quantitative analysis of tumor signals between Np17 KD and control(p<0.001). (**G**) overall survival (Kaplan-Meier analysis).

Given that *np17* is a cellular homolog of viral *np9* oncogene, to determine whether Np17 is essential for the survival and growth of leukemia cells, we next determined whether knockdown of Np17 (Np17-KD) affect survival of leukemia cells. Recombinant lentiviruses transcribing short hairpin RNAs against Np17 were generated and transduced to Np17-expressing human THP-1 and NB-4 leukemia cells. MTT assay was used to monitor survival and proliferation of leukemia cells. We observed that Dox-induced knockdown of Np17 markedly inhibited the growth of THP-1 leukemia cells (Figure 5A, 5B). We next examined the effects of Np17-KD on the colony-forming ability of leukemia cells using colony-forming assay and found that knockdown of Np17 resulted in a significant decrease in the number of colonies as compared with control (Figure 5C, 5D). Consistently, Dox-induced knockdown of Np17 also inhibited the growth and colony formation of NB-4 (AML-M3) leukemia cells (Supplementary Figure 4A–4C).

Because no cellular homolog of Np17 was found in mice, it is infeasible to carry out knock-in or knock-out of Np17 in mice. To evaluate the effect of Np17 on survival and growth of leukemia cells *in vivo*, we established orthotopic human AML model in NSG mice using AML THP-1-luciferase cells with Dox-inducible Np17-KD vector or control vector. Briefly, 2x10^6^ THP-1 cells with Dox-inducible Np17-KD vector (Np17-KD) or empty vector (Control) were injected through the tail vein into NSG (NOD/SCID/IL2Rγ-/-) mice. After detecting obvious tumor signal (day 27 after cells injection), mice received Dox to initiate Np17 knockdown via oral gavage. Consistent with the *in vitro* results, Np17-KD induced a significant growth inhibition of leukemia cells in NSG mice as compared with controls. A 4.2-fold decrease of tumor signal was observed with Np17 knockdown compared with control at 8 day after Np17-KD initiation (Figure 5E, 5F). Consistent with these results, Np17-KD prolonged overall survival of recipient mice as compared with control mice (Figure 5G, *P*=0.006).

### Np17 promotes the growth of leukemia cells

To determine whether Np17 promotes the growth of leukemia cells, we generated lentiviral expression constructs for Np17 and introduced them individually into various human leukemia cell lines MOLM-13 and K562. We observed that at 7^th^ day, the cell number in the Np17-expressing MOLM-13 was 1.94-fold higher than that of control (*P*<0.001) (Supplementary Figure 5A, 5B). Consistently, colony formation assay also showed that the Np17 expression led to a significant increase by 2.48-fold in the number of colonies (Supplementary Figure 5C, 5D). Similar results were observed in K562 leukemia cells (Supplementary Figure 6).

To evaluate the tumor promotion potential of Np17 overexpression *in vivo*, Np17-overexpression (Np17-OE) K562 cells or control cells were engrafted subcutaneously into NOD/SCID mice and the tumor signal was monitored every 7 days. The tumor volume and weight in the Np17-overexpressing cells were 4.86-fold and 2.63-fold increases, respectively, as compared with control cells (Supplementary Figure 7A–7C) at day 20. These data indicated that Np17 plays critical roles in maintaining survival and growth leukemia cells *in vitro* and *in vivo*.

### Np17 reduces stress-induced P53 protein

**Figure 6:**
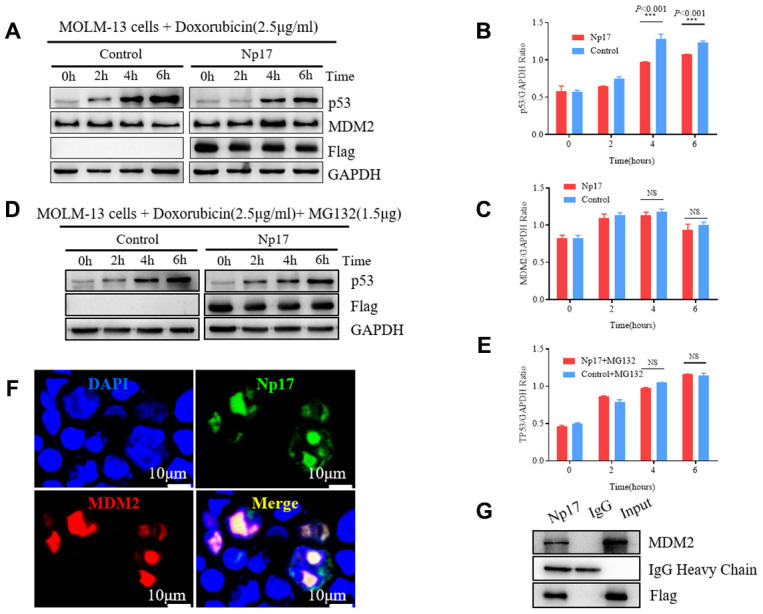
**Np17 induces proteasome-dependent degradation of p53 by recruiting MDM2.** (**A**–**C**) Western blot showed that Np17 expression inhibited doxorubicin-induced p53 activation but did not affect MDM2. The topoisomerase II inhibitor doxorubicin (2.5 μg/mL) to induce p53 for the indicated times. (**D**, **E**) Proteasome inhibitor MG132 attenuated Np17-mediated P53 degradation. (**F**) EGFP-Np17 protein (Green) is co-localized with MDM2 (Red) in the cell nucleus of HEK293 cells. (**G**) Cell lysates were immunoprecipitated (IP) with Flag antibody or control IgG, and the immunoprecipitates were analyzed by Western blot analysis with MDM2 antibody.

The viral *np9* gene has been shown to the P53-MDM2 pathway [38], which is frequently dysregulated in AML [39–42]. P53, a critical transcription factor that inhibits cell division or survival in response to various stresses such as DNA damage, and acts as a key fail-safe mechanism of cellular anti-cancer defenses, is central to hematopoietic stem cell functions and its aberrations affect AML evolution, biology, and therapy response [40], whereas MDM2 is the major E3 ubiquitin ligase for P53 degradation. Thus, we next investigated whether Np17 plays a role in P53-MDM2 pathway. We expressed Np17 protein in human MOLM-13 leukemia cells (in which the P53 protein was wild-type), and looked for the effects of Np17 on p53 and MDM2 protein levels after stress by doxorubicin, a common chemotherapeutic agent that activates P53. We observed that Np17 overexpression reduced doxorubicin stress-induced P53 protein level, but did not affect MDM2 in leukemia cells (Figure 6A–6C). Moreover, we found that co-treatment with a proteasome inhibitor, MG-132, could rescue the Np17-induced decline in P53 protein (Figure 6D, 6E). To further explored whether MDM2 was involved in Np17-mediated TP53 protein reduction, we performed co-IP experiment and found that that Np17 physically interacted with MDM2 (Figure 6G). Co-localization experiments showed that Np17 and MDM2 were co-localized with P53 protein in the nucleus of HEK293 cells (Figure 6F). These results suggest that Np17 may decrease P53 levels by recruiting nuclear MDM2 to P53 for ubiquitin-mediated degradation in cells.

## DISCUSSION

In this study, we have identified a novel oncogene *np17 gene,* a cellular homolog of the viral *np9* gene, in human leukemia cells. This novel gene, which is aberrantly activated in human acute myeloid leukemia patients and associated with relapse/refractory AML, has important roles in survival, and proliferation of leukemia cells. Our studies also reveal that Np17 may decrease P53 levels by recruiting nuclear MDM2 to P53 for ubiquitin-mediated degradation in cells, which is frequently dysregulated in AML [39–42].

It is well known that a number of cellular oncogenes are derived from retroviruses such as Myc, Ras and Src [21–35]. Because our previous studies defined viral *np9* gene as a potent oncogene, which is aberrantly activated in a majority of human leukemia, and promotes the growth of leukemia stem/progenitor cells by co-activating multiple cancer-related signaling pathways, we therefore speculated that cellular homologs of this viral oncogene may exist in human leukemia cells. As expected, we identified *np17* as a cellular homolog of the viral *np9* gene in human leukemia cells by performing alignment using Np9 protein sequence, cloning and sequence analysis. We demonstrate that the *np17* gene is located on chromosome 8p23.1 and contains 7 exons. The 1-4 exons are non-coding exons, and the exons 5-7 encode the nuclear Np17 protein. Interestingly, we found that this novel gene is predominantly present in Hominoidea as the *np9* gene. This result support our hypothesis that *np17* was a homolog of the *np9*.

Next we investigated the expression of Np17 in AML patients. The results showed that the levels of Np17 mRNAs were significantly higher in primary AML patients than those in normal controls. And 59.65% of AML patients exhibited higher Np17 mRNAs as compared with normal control. Consistently, the Np17 protein could be detected in some AML samples, but low or absent in normal hematopoietic stem cells. Importantly, Np17 expression is highly activated in 22.81% of leukemia patients and high levels of Np17 might be a high risk factor for AML patients.

Functionally, our studies showed that Silencing Np17 led to growth inhibition of leukemia cells, whereas overexpressing Np17 promoted growth of various leukemia cells *in vivo* and *in vitro*. These findings indicate that Np17 plays important roles in survival and growth of leukemia cells.

Mechanistically, our observations revealed that Np17 decreased doxorubicin stress-induced P53 protein level, but did not affect MDM2 in leukemia cells. Moreover, co-treatment with a proteasome inhibitor could rescue the Np17-induced P53 decrease and Np17 physically interacted with MDM2. Given that P53-MDM2 pathway is frequently dysregulated in AML (39-42), we hypothesize that Np17 may play a critical role in dysregulated P53 of AML, and its mechanism may be involved in recruiting nuclear MDM2 to P53 for ubiquitin-mediated degradation in cells.

In summary, our findings reveal that *np17* is a novel oncogene that promotes the growth of leukemia cells, and its mechanism may be associated with negative regulation of P53 in AML. Further studies of Np17 are needed to insights into the detailed mechanisms by which Np17 regulates survival and growth as well P53 degradation of leukemia cells. These are under investigation.

## MATERIALS AND METHODS

### Preparation of Np17 antibody

The Np17 rabbit antibody was prepared in our lab. To raise rabbit polyclonal antibodies, the cDNA of recombinant Np17 with 6xHis in C-terminal was cloned into the Pet-28a plasmid. After induced with IPTG, the whole protein lysate was purified using Ni-NTA resin affinity chromatography and was eluted with 300mM imidazole. And then, the recombinant Np17 protein was confirmed by Western blot with His-antibody (α-His) and Np9-antibody (α-Np9). Rabbits were immunized with the purified recombinant Np17 protein for four times. After that, the serum antibodies of the rabbits were purified with affinity chromatography. The Np17 antibody dilution for Western blot was 1:1000-1:5000 and its specificity was confirmed by the recombinant Np17 protein blocking assay.

### Reagents and antibodies

Doxorubicin, Blasticidin S, puromycin and DMSO were purchased from Sigma-Aldrich. Anti-GAPDH (5-E10) and the horseradish peroxidase (HRP)-conjugated secondary antibodies were obtained from HuaAn Biotechnology Co., Ltd. Antibodies against His, Flag, MDM2, p53 and Lamin A/C were from HuaAn Biotechnology Co., Ltd.

### Cell lines and culture

Seven various human hematopoietic malignant cell lines were used in these studies, included KASUMI-1, KG-1a, AML-3, THP-1, MOLM-13, NB-4, HL-60. Cells were cultured in RPMI-1640 supplemented with 10% fetal Bovine serum (FBS) at 37°C in a 95% air, 5% CO_2_ humidified incubator.

### Human leukemia cell samples and normal blood samples

Human primary leukemia cell samples and normal blood cell samples were obtained from leukemia patient tissue bank and healthy volunteers with their informed consent in accordance with the Declaration of Helsinki. Granulocyte colony stimulating factor mobilized bone marrow stem cell samples were obtained as residual material from normal donors for allogeneic transplantation. All experiments were approved by the ethics committee of Second Affiliated Hospital, School of Medicine, Zhejiang University (IR2018001163).

### Isolation mononuclear cells and CD34+ cells

Isolation mononuclear cells were obtained from healthy peripheral blood donors and bone marrow cells of AML primary samples by density gradient centrifugation using Ficoll reagent. CD34+ cells were isolated by positive selection for the cell surface marker CD34 using MojoSort™ Nanobeads (BioLegend) in accordance with the manufacturer's instructions.

### Lentivirus packaging

The HEK293T cells were cultured in the 10cm dish for 24 hours before lentivirus packaging. The 9μg target plasmid was co-transfected into the HEK293T cells with 3μg psPAX2, 3μg pMD2.G and 40μL polyjet. The supernatant was harvested in 48 hours and 72 hours after transfection. After ultrafiltration, the supernatant contained lentivirus was titrated. The cells were transduced with the lentivirus with an optimal MOI.

### Generation of EGFP-Np17 and EGFP-Np17-ΔNLS cells and subcellular localization assay

The double-strand Np17-ΔNLS DNA was synthesized by Huabio. The Np17 cDNA and Np17-ΔNLS DNA were cloned into pEGFP-N1 vector (digested with EcoRI and BamHI), respectively, with an EGFP tag at the N-terminus of Np17. For subcellular localization assays, HEK293 cells were transfected with EGFP-Np17 plasmid, EGFP-Np17-ΔNLS plasmid and EGFP plasmid for 48 hours respectively. Cell nuclei were counterstained with DAPI (blue) followed by observation under Zeiss Confocal Laser Scanning Microscope 710.

### Generation of EGFP-Np17 and MDM2-mCherry cells and co-localization assay

The MDM2 cDNA was cloned into pLVX-EF1α-mCherry-N1 vector with EcoRI and BamHI digested. This pLVX-EF1α-MDM2-mCherry-N1 plasmid and the pEGFP-Np17 plasmid (generated above) were co-transfected to the HEK293 cells for 48 hours. Cell nuclei were counterstained with DAPI (blue) followed by observation under Zeiss Confocal Laser Scanning Microscope 710.

### Construction of Np17 shRNA lentivirus and Np17-KD cells

The three shRNAs targeted 5’-GCCATCTTCATCATTGCATTA-3’, 5’-GCATGCCTGCCATCTTCATCA-3’, 5’-GCAATGATGAAGATGGCAGGC-3’ in the message RNA sequences of Np17. The double-strand DNAs were synthesized by Huabio and then cloned into the tet-pLKO-puro (Addgene, #21915), which had been digested by EcoRI and AgeI. The lentivirus was packaged as the procedure above. THP-1 and NB-4 cells were transduced with the lentivirus with a MOI of 10 and selected by puromycin for 10 days.

### Construction of Np17 expression plasmid and Np17-OE cells

The 3xFlag-Np17 DNA was ligated into the pLVX-EF1α-mCherry-N1 plasmid which has been digested by EcoRI and BamHI. The lentivirus was packaged as the procedure above. MOLM-13 cells were transduced with the lentivirus with a MOI of 10 and selected by puromycin for 10 days.

### Cell proliferation assay

Cell proliferation was determined by the methyl-thiazol-tetrazolium (MTT) assay. Briefly, 7.5×10^2^ cells/well were seeded in 96-well plates. Cells were cultured for 0,1,3,5 and 7 days, respectively. After cultured, MTT (Chemicon, Temecula, CA) was added to each well and incubated at 37°C for 4 hours. Cells were then lysed by adding 0.1mL lysis solution. Absorbance was measured at 562nm by using a microplate reader and reported as optical density (OD) after incubated at 37°C for 12 hours.

### Colony formation assay

Cells were cultured in a 6-well plate using 1640 medium with 15% fetal calf serum (FCS) at 37°C in a 95% air, 5% CO_2_ humidified incubator for 21 days. The initial numbers of cells in the plate were 1000/well. Each well of cell colonies were scored (≥40 cells).

### Western blot

Cells were collected and lysed in protein extraction reagent (78501, Thermo scientific) containing protease and phosphatase inhibitor (1861281, Thermo scientific). The proteins were separated in SDS-page and were transferred to PVDF membranes (Bio-Rad). The bound antibodies were visualized using Super signal reagents (Thermo Fisher Scientific).

### Blocking assay

The Np17 antibody was pre-incubated with recombined Np17-6xHis protein used for immunization at 4-times concentration at 4°C overnight and then used to detect Np17 proteins from a primary leukemia cell sample using Western blot.

### Extraction of nuclear Np17 protein

2x10^6^ cells of THP-1-Np17-KD and THP-1-Control cells (both generated above) were collected with 500g horizontal centrifugation for 5 minutes. Both of the cells were incubated with 150μL cytoplasmic lysis buffer (YM-017, Invent) at 4°C for 5 minutes. The lysates were centrifuged with 14000g at 4°C for 5 minutes. After wash with 1x PBS, both of the precipitates were incubated with 50μL nuclear lysis buffer at 4°C for 30 minutes and centrifuged with 14000g for 15 minutes to generate nuclear protein.

### Real time-PCR analysis (qRT-PCR)

Total RNA was extracted by using Trizol (Invitrogen) according to the manufacturer’s instructions. 500ng of total RNA was reverse transcribed to cDNA using PrimeScriptTM II 1^st^ strand cDNA Synthesis Kit (Takara). qRT-PCR were performed with SYBR® Premix Ex TaqTM II (Tli RNaseH Plus) (Takara) on 7500 Real-Time PCR Systems (Applied Biosystems, USA). Gene expression level was determined by using the ΔΔcycle threshold method normalized to β-Actin. qPCR primers used were as follows: Np17 forward, 5’-GAAGGCAGCCCTTTTCTAGATG-3’; Np17 reverse, 5’- CCCTCCAGGAGTTTACATGAG-3’; ACTIN forward, 5’-ACTCTTCCAGCCTTCCTTCC-3’; ACTIN reverse, 5’-AGCACTGTGTTGGCGTACAG-3’. All experiments were performed in triplicate in a 20μl reaction volume.

### Effect of Np17 on TP53 protein

1x10^6^ MOLM-13-3xFlag-Np17-OE cells per well or MOLM-13-Control cells (both generated above) were seeded in 6-well plate. 2.5μg/mL doxorubicin was added in each well in the presence or absence of 1.5μM MG132 and then cultured for 0,2,4,6 hours. The cells were harvested for analysis of Np17 and p53 protein by Western blot. Quantitative analysis of Western blots was carried out using the Image J software.

### Co-immunoprecipitation analysis of Np17 and MDM2

1x10^8^ MOLM-13-3xFlag-Np17-OE cells were collected and lysed with 800μL of NP40 lysis buffer in 1.5mL-Eppendorf tube. And then the lysate was divided into two groups equally. 3μg Flag antibody and 3μg control IgG were added into the two groups respectively. Each group was incubated in 4°C for 12 hours, and then was incubated with 20μL protein G magnetic beads in 4°C for 16 hours. After incubation, the supernatants were removed and the beads were washed with 1mL of NP40 lysis buffer for 4 times. The Co-IP proteins were eluted with loading buffer and followed by Western blot analysis.

### *In vivo* study of effect of Np17 knockdown

The lentivirus was packaged as the procedure above. 2x10^6^ THP-1 cells were transduced with the lentivirus of luciferase with a MOI of 10 and were selected by blastcidin with 15μg/μL for 2 weeks. These luciferase-THP-1 cells were divided into two groups, and were transduced with the lentivirus of Np17-KD and control respectively as above. After selected by puromycin, the two groups of cells were prepared for *in vivo* analysis. 2x10^6^ luciferase-THP-1 cells with Dox-inducible Np17-KD vector or control cells were injected into female NSG mice (8-weeks) through the tail vein. After detecting obvious tumor signal by bioluminescence imaging using an IVIS 100 bioluminescence/optical imaging system (Xenogen, Alameda, CA, USA), mice received Dox to initiate Np17 silencing via oral gavage.

### NOD/SCID mouse tumor xenograft with K562 cells with Np17 lentivirus vectors

To generate K562 stable cell line that express Np17, the protein coding sequence was cloned into a lentiviral vector with MSCV promoter (5’UTR) and puromycin resistance gene. The vectors were then used to package lentivirus and infect the K562 cells with a MOI of 3. The K562 cells were then selected with puromycin for 1 week and used for xenograft. Briefly, 1 x 10^7^ cells (K562 with NP17 overexpression, K562 with control vector) were inoculated subcutaneously in the right and left flanks of NOD/SCID female mice, respectively. Tumor volume and mouse body weight were measured at different time points. At the end of experiments, all mice were euthanized for analysis of body weight and tumor weight.

### Animal studies

Animal studies were approved by the Zhejiang Chinese Medical University Animal Care and Welfare Committee (#ZSLL-2017-067).

### Evolutionary relationships of *Np17*

The evolutionary history was established using the Neighbor-Joining method. The optimal tree with the sum of branch length = 3.26688660 is shown. The percentage of replicate trees in which the associated taxa clustered together in the bootstrap test (500 replicates) is shown next to the branches. The tree is drawn to scale, with branch lengths in the same units as those of the evolutionary distances used to infer the phylogenetic tree. The evolutionary distances were computed using the Poisson correction method and are in the units of the number of amino acid substitutions per site. The analysis involved 4 amino acid sequences. All positions containing gaps and missing data were eliminated. There were a total of 75 positions in the final dataset.

### Statistical analysis

All data are presented as mean values ± SEM. Statistical analysis (two-tailed t-test, Pearson correlation, log-rank test and Tukey's multiple comparison test) were performed using Prism 6 (GraphPad Software). Differences with *p value* < 0.05 were considered statistically significant. Differences are labeled as follows: * for *p* < 0.05; ** for *p* < 0.01; *** for *p* < 0.001.

## Supplementary Material

Supplementary Figures

Supplementary Tables
