# Supplementary material for: Identification of the novel Np17 oncogene in human leukemia
Source: Aging (Albany NY). 2020 Nov 21;12(23):23647–67. doi: 10.18632/aging.103808 (PMC7762455; doi:10.18632/aging.103808)
Supplement: Supplementary Figures [file aging-12-103808-s001.pdf]

SUPPLEMENTARY FIGURES

ACGGGGAGGGGTTTGAGGAGGGGATGGGAATGTGGCTCAAGTTCGGGAGGCGTTACCTGCGGAGGGTTTGAGGCAGGCCCAGGAGCGAGC
CCATGGTCTTCCGCCGCGGGGCCAGGGGCGGGCCCCAGGATCCGGAGCTTCGTGCGGGGCCGAGTCCAGGTTTGGGGCCCGGGAGGCGGGG
CCAGTTAGGGCGAGGGTCCCTGGGATCGTCGGGTCAGGCCTTGGGCTAACGTAGGCACTCTCGCAGTTCCTCCGCCTTCAGGAAGGTCTTTT
CAGCAGGGGCCTTACGGGTGCATGCTTCGGTCCTGGAGGCCTTATCCTAGCCTCCTCTCCATCAGCGCCACCCGTCTGGGGCCCGAAAGGAG
GGAGCTTTCCCTCTGTCCCCCAGCCTTTGGACTGTCAGCAAACAAGCCATTCGTTCATCAAATACTTTTTAAGCGCCTACCATGTGCCTGACA
AGGGAGATGTAACGGTGAGAAAAACTAGGTGTGGTCCAGGCCCTTCAGGGGCTCAGGTGCTCGTGGAAGAAGTGGACATTGAAGTACTTAT
CACACAAATGAGGATAAAAGTACGATAGCGATATCTACCACGAAGCTGTTGTTCCCCACCAGAACCAAATGAGCGCAAGATCTGACAAAGA
AAAAAAAAGGTTCATCTTTTATTCCTCCAAACACTTTCATTTAAATCAAGAGGATGGGATGTGGTTATTGCTGTGTTTTAGACAGAATCAAC
GGTTTCTGGGTCTGAGATGTTGCATACACCTTCTCAGTCCCTGTATCCTGAGATGGAGTCACCTGAGAATCCACAGCAAGTCCTAACCAGGG
ATGGGTCTGGGTGATTAAGGAAGGTTGGCTTCAGAACTGGGCCAGGGGCACTGCTTTGCTTTTGCTGTTTTGATCAGCTCTCTGCCTGAAGG
AGACAAGAAAAACCAACGGAACAGCTGGGGAAACTGAGTTACAGAGCTTGTCTGCACTGAGTCATCAGGAGCAAATGCTAGATCAGATA
GGGTCTCTCTCTCTGCCACCCAGGCCAGAGTGCAGTTGACGCAGGGCAGGGGAGCCCCGAAGTGGAGCATAGTGTGTCCGGAACTGGTGGG
TTCTTGGTCTCACTGACTTCAAGAAAGAAGCCGCGGACCCTCGCGGTGAGTGTCACAGTTCTTAAAGGCTGCGTGTCCAGAGTTTGTTCCTTC
TGATGCTCGGATGTGTTCAGAGTTTCTTCCTTCTGGTGGTTTTGTGGTCTCGCTGGCTTCAGGAGTGAAGCTGCAGACCTTCAAGAA**ATG**AGG
CTTCTCCCCAGGACCAGCATTCCACGAAGGCAGCCCTTTTCTAGATGGAGAAAACAGAACCTGAAGACACCCGTTTCCCTAAACTGCTCTCG
CTCATGTAAACTCCTGGAGGGCAGAGGCATGCCTGCCATCTTCATCATTGCATTACCACCACCCAACACTATCGGGGGACCTGCCCTGATAA
TCAGTCTACAGGTGTATCCAGCAGCTCCAGAGAGACAGCGACCAGCGAGAAGGGGCCATGATGATGGAGGTGGTTTTGTCAAAACGAAAAT
GGGGATATGTAGGGAAAAGAAAGAGAGATCAGACTGTTACTGTGTCTACATAGAAAGGGAAGACATAAGAGACTCCATTTTGAAAAAGAC
CTGTACTTTAAACAATTGCTTTGCTGAGATGTTGTTAATCTGTAGCTTTGCCCCAGCCACTTTGCCCCAACCACTT**TGA**CCCAATCTGGAGCT
CATAAAAACATGTGTTGTATGAAATCAAGGTTTAAGGCATGTAGGGCTGTGCAGGACGTGCCTTGTTAACCAAATGTTTGCAAGCAGTATAC
TTGGTAAAAGTCATCACCATTCTCTCGTCTCAATAAACCAGGGGCACAATGCACTGTGGAAAGCCGCAGGGACCTCTGCCCTTGAAAGCTGG
GTATTGTCCAAAGTTTCTCCCCATGTGATAGTCTGAAATATGGCCTCGTGGGATGAGAAAGACCTGACGGTCCCCCAGCGCGACACCCATAA
AAGATCTGTGCTGAGGTGGATTAGTCAAAGAGGAAAGACTTGCAGTTGAGATAGAGGAAGGCCACTGTCTCCTGACTGCCCCTGGGAACTG
AATGTCTCGGTATAAAACACGATTGTACATTTGTTCAGTTCTGAGATGGGAGAAATACCGCCCTATGGTGGGAGGCGAGACATGTTTACAGC
AATGCTGCCTTGTTATCCTTTACTCCACTGAGATGTCTGGGTGGAGAGAAACATAAATCTGGCTTACGTGCACGTCCAGTCATAGTACCTTCC
CTTGAACTTCATTATGTCATAGATTCTATTGCTCACGTTTGTTGCTGACCTTCTCCTTATTATCACCCTGCCCTCCTACTACATTCCTTTTTGCT
GAAATAATGAAGATAATAATCAATAAAAACTGAGGGAATTCAGAGACCTGTGCCAGTGCAGGTCCTTAGCATGCTAAGCGCAGGTCCCCTG
GGCCCGCTGTTGTTTTTTTTT

Supplementary Figure 1. Sequences of Np17 mRNA and open reading frame (ORF). Np17mRNA: gi|34526700|dbj|AK129982.1|, Homo sapiens cDNA FLJ26472 fis, clone KDN04506 (2501bp). ORF sequence is highlighted by blue color.

A

```
Human Np17         1   MRLLPRTSIPRRQPFSRWRKQNLKTPVSLNCSRSCKLLEGRGMPAIFIIALPPPNTIGGP   60
                       MRLLPRTSIPRRQPFSRWRKQNLKTPVSLNCSRSCKLLEGRG PAIFIIALPPPNTIGGP
Chimpanzee Np17    1   MRLLPRTSIPRRQPFSRWRKQNLKTPVSLNCSRSCKLLEGRGRPAIFIIALPPPNTIGGP   60

Human Np17         61  ALIISLQVYPAAPERQRPARRGHDDGGGFVKTKMGICREKKERSDCYCVYIEREDIRDSI   120
                       ALIISLQVY AAPERQRPARRGHDDGGGFVKTKMGICREKKERSDCYCVYIEREDIRDSI
Chimpanzee Np17    61  ALIISLQVYSAAPERQRPARRGHDDGGGFVKTKMGICREKKERSDCYCVYIEREDIRDSI   120

Human Np17         121 LKKTCTLNNCFAEMLLICSFAPATLPQPL   149
                       L+KTCTL NCFAEMLLICSFAPATLPQPL
Chimpanzee Np17    121 LEKTCTLKNCFAEMLLICSFAPATLPQPL   149
```

B

```
Human Np17     1   MRLLPRTSIPRRQPFSRWRKQNLKTPVSLNCSRSCKLLEGRGMPAIFIIALPPPNTIGGP   60
                   MRLLPRTSIPRRQPFSR RKQNLKTPVSLNCSRSC
Gorrila Np17   1   MRLLPRTSIPRRQPFSRQRKQNLKTPVSLNCSRSC-------------------------   35

Human Np17     61  ALIISLQVYPAAPERQRPARRGHDDGGGFVKTKMGICREKKERSDCYCVYIEREDIRDSI   120
                        LQVYPAAPERQRPARRGH+DG GFVK K GICREKKE  DCYCVYIE+EDIRDSI
Gorrila Np17   36  -----LQVYPAAPERQRPARRGHNDGAGFVKKKRGICREKKE--DCYCVYIEKEDIRDSI   88

Human Np17     121 LKKTCTLNNCFAEMLLICSFAPATLPQPL   149
                   LKK CTLNN FAEMLLICSF PA+L QPL
Gorrila Np17   89  LKKACTLNNFFAEMLLICSFTPASLTQPL   117
```

Supplementary Figure 2. Amino acid sequence alignments of Np17. (**A**, **B**) Amino acid sequence alignments of human Np17 with Chimpanzee (**A**) and Gorrila (**B**) Np17.

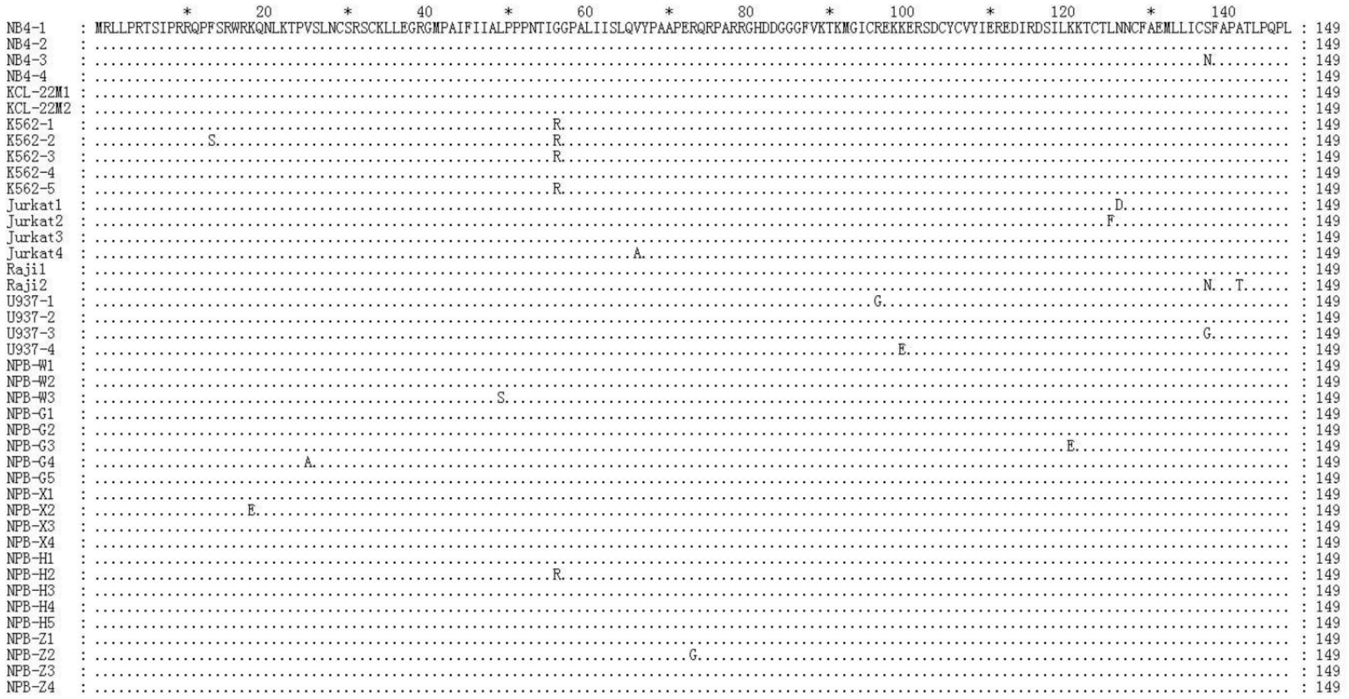

Supplementary Figure 3. AA alignment of Np17 proteins of 42 cDNA clones from various leukemia cell lines and normal individuals.

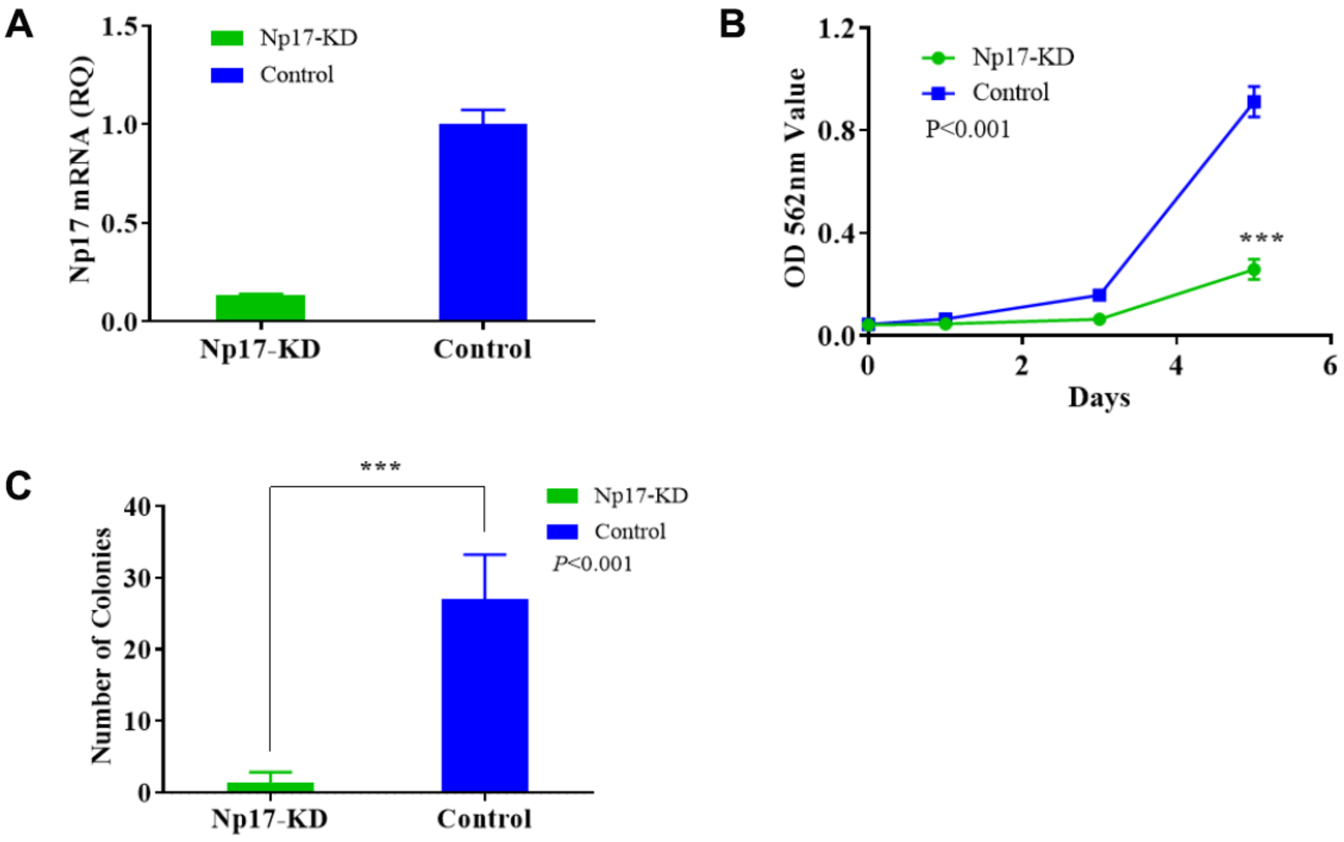

Supplementary Figure 4. Knock-down of Np17 inhibits growth of leukemia cells. (**A**) qRT-PCR showed that shRNA reduced np17 mRNA levels. (**B**) Np17-KD inhibited the growth of NB4 cells. (**C**) Np17-KD decreased colony numbers of NB4 cells.

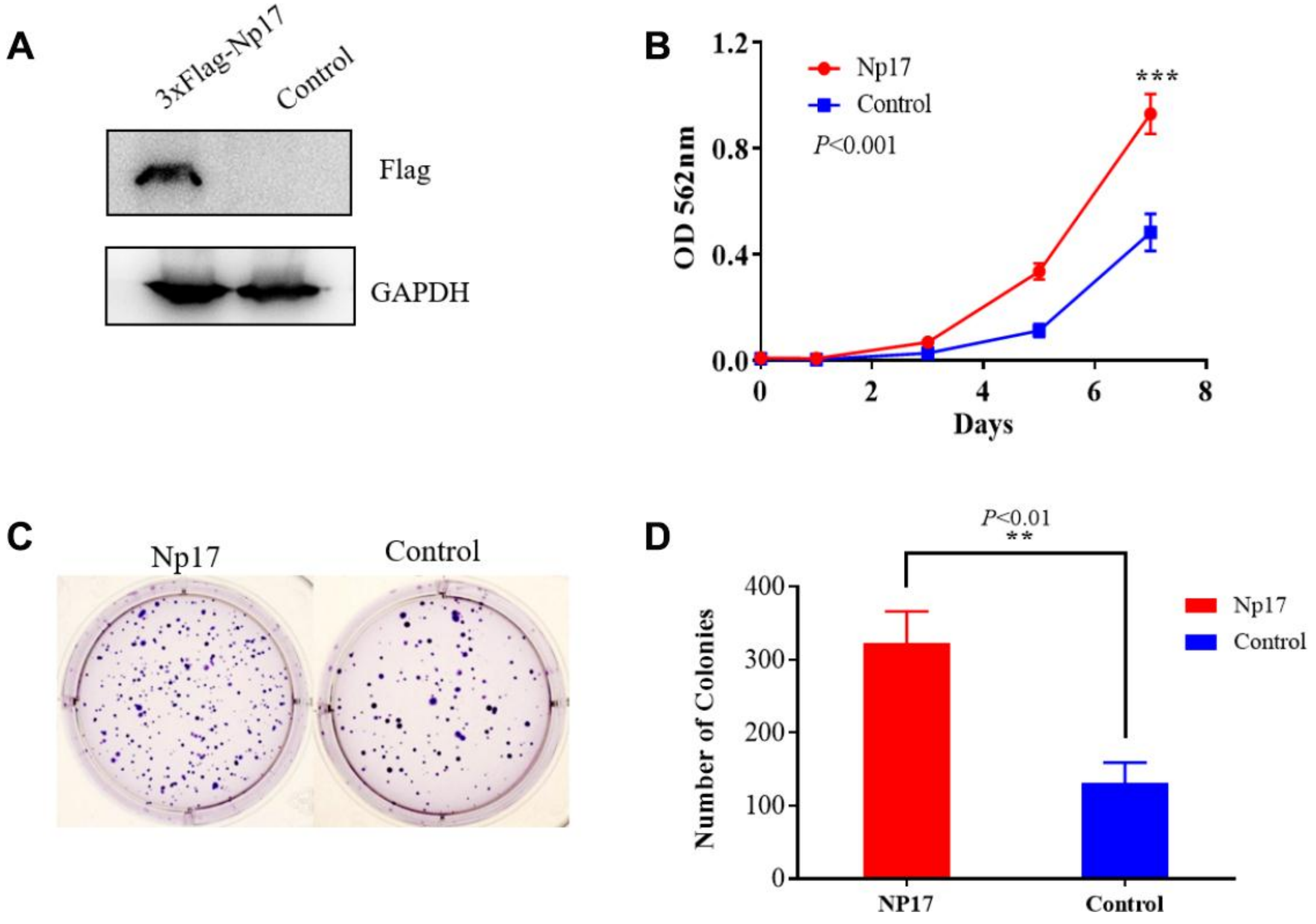

Supplementary Figure 5. Overexpression of Np17 promotes the growth of AML cells. (**A**) Western blotting analysis of Np17 levels in MOLM-13 cells after Np17 overexpression (Np17-OE). (**B**) Comparison of proliferation curves of Np17 overexpression with control in MOLM-13 cells. (**C**, **D**) Representative images and quantification of colony numbers of Np17-OE compared with control in MOLM-13 cells.

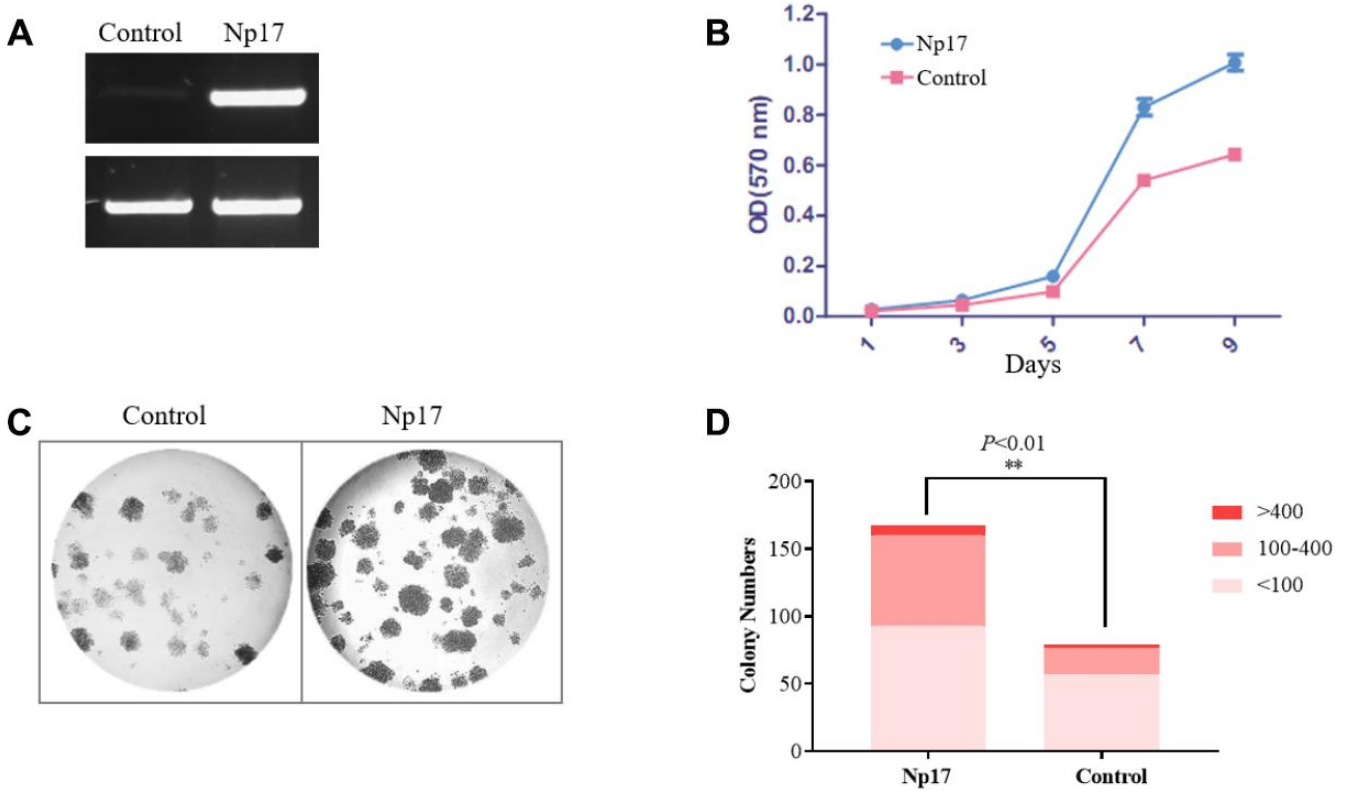

Supplementary Figure 6. Overexpression of Np17 promotes proliferation and colony forming potential of tumor cells of CML blast crisis. (**A**) PCR analysis of Np17 levels in K562 cells. (**B**) Comparison of proliferation curves between Np17- overexpressing K562 cells and control cells. (**C**, **D**) Representative images and quantification of colony numbers in K562 cells Np17-OE compared with control.

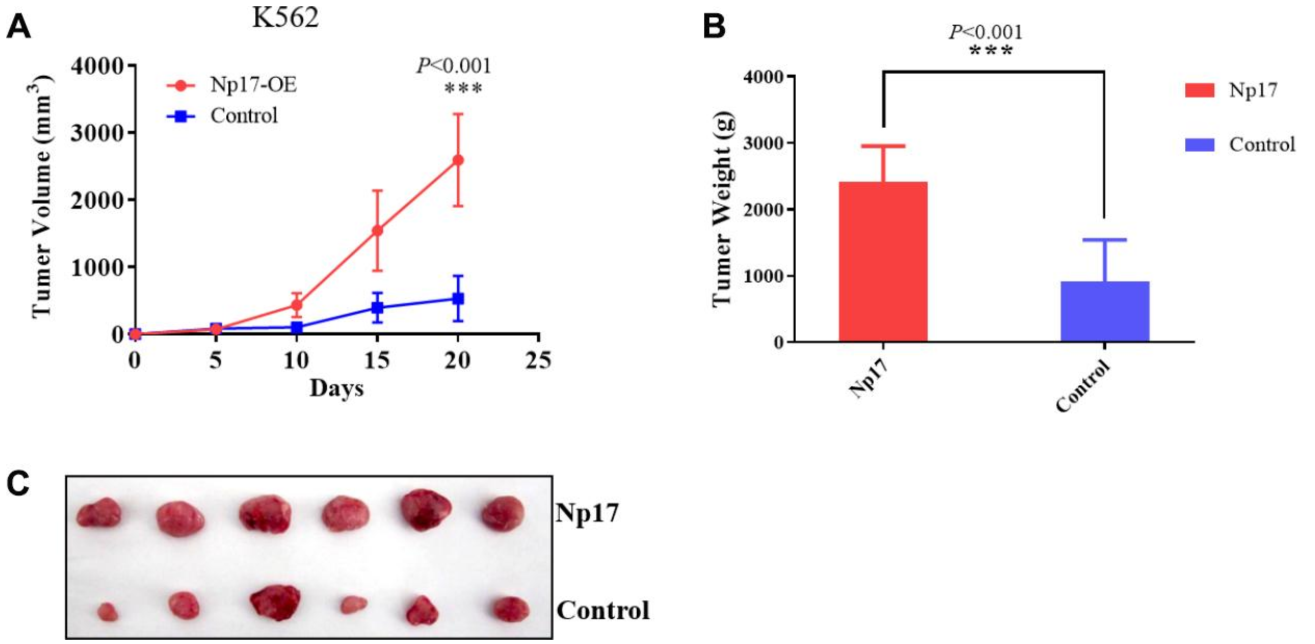

Supplementary Figure 7. Overexpression of Np17 promotes the growth of leukemia cells in nude mice. (**A**) Tumor volume comparison between Np17-OE and control (n=6). (**B**) Tumor weight comparison between Np17- OE and control at the end of experiments (n=6). (**C**) Representatives images of xenografts of Np17-OE and controls.
