# Supplementary material for: Identification of the novel Np17 oncogene in human leukemia
Source: Aging (Albany NY). 2020 Nov 21;12(23):23647–67. doi: 10.18632/aging.103808 (PMC7762455; doi:10.18632/aging.103808)
Supplement: Supplementary Tables [file aging-12-103808-s002.pdf]

SUPPLEMENTARY TABLES

Supplementary Table 1. The clinical data of the patients involved in the Western Blotting analysis.

No.	FAB Classification	Gender	Age (y)	WBC ($x10^9$/L)	BM blast cells (%)	PLT ($x10^{12}$/L)	Hb (g/L)
1	AML-M2	Male	15	3.1	20.0	33.0	80.0
2	AML-M2	Female	70	8.6	81.0	80.0	90.0
3	AML-M2	Male	23	78.8	83.0	19.0	108.0
4	AML-M4	Female	41	98.9	58.0	14.0	151.0
5	AML-M5	Male	51	22.8	32.5	118.0	72.0
6	AML-M1	Female	58	4.2	68.0	24.0	64.0
7	AML-M5	Male	17	26.5	41.7	35.0	67.0

AML, acute myeloid leukemia; WBC, white blood cells; BM, bone marrow; PLT, platelet; Hb, hemoglobin.

Supplementary Table 2. The clinical data of the patients involved in the qRT-PCR analysis.

No.	FAB Classification	WBC ($x10^9$/L)	BM blast cells (%)	PLT ($x10^{12}$/L)	Hb (g/L)	Np17-RQ	RR AML
1	AML-M2a	49.0	92.8	67.0	64.0	0.73	No
2	AML-M2a	17.3	60.5	10.0	76.2	5.65	No
3	AML-M4eo	154.6	NA	26.0	71.0	0.79	Yes
4	AML-M4b	9.3	60.0	40.0	114.4	0.33	NA
5	AML-M5	284.9	67.5	27.0	72.0	0.56	NA
6	AML-M5b	248.6	92.5	127.0	106.4	0.91	No
7	AML-M2a	113.8	20.0	32.0	60.2	2.69	Yes
8	AML-M0	199.5	96.9	17.0	69.0	0.87	NA
9	AML-M2a	12.8	42.5	173.0	78.8	0.27	Yes
10	AML-M2a	53.6	47.5	118.0	62.0	7.67	No
11	AML-M1	131.2	89.0	59.0	3.0	0.72	NA
12	AML-M5	15.2	73.5	62.0	76.8	1.27	Yes
13	AML-M1	259.3	91.0	21.0	95.0	1.27	Yes
14	AML-M4	4.9	69.0	294.0	131.0	0.33	Yes
15	AML-M2a	51.8	70.0	46.0	94.0	0.74	NA
16	AML-M5	116.2	75.5	16.0	56.0	1.24	NA
17	AML-M4b	208.6	50.5	36.0	68.0	0.20	NA
18	AML-M1	3.2	90.5	473.0	95.0	0.34	No
19	AML-M5	189.5	46.0	58.0	89.0	0.29	NA
20	AML-M2a	NA	75.0	NA	NA	0.60	No
21	AML-M1	222.5	90.0	33.0	94.0	0.62	NA
22	AML-M2	98.8	54.0	17.0	95.5	1.27	Yes
23	AML-M2a	11.4	65.0	17.0	139.0	0.27	Yes
24	AML-M1	5.1	96.0	180.0	74.0	3.44	No
25	AML-M5b	80.3	77.0	47.0	81.0	0.17	NA
26	AML-M5b	66.8	69.0	115.0	68.0	0.08	NA
27	AML-M2a	11.4	72.5	35.0	64.0	0.88	No
28	AML-M0	2.5	92.0	234.0	111.2	1.83	Yes
29	AML-M5b	173.3	77.0	53.0	101.2	0.36	Yes
30	AML-M3	20.6	93.0	16.0	150.0	6.33	Yes
31	AML-M2a	0.6	73.5	48.0	85.0	13.79	No
32	AML-M2a	1.5	23.0	20.0	70.4	4.24	No
33	AML-M4	192.8	33.0	16.0	96.0	0.36	Yes
34	AML-M2a	22.6	73.0	10.0	63.0	0.71	No
35	AML-M1	21.9	75.0	22.0	100.7	1.18	Yes
36	AML-M2a	56.5	69.4	25.0	135.0	1.04	No
37	AML-M2a	63.1	88.5	18.0	77.4	9.10	NA

38	AML-M5b	30.9	54.0	28.0	37.4	2.25	No
39	AML	NA	NA	NA	NA	12.45	NA
40	AML-M2a	214.0	88.0	778.0	104.0	3.66	No
41	AML-M5	200.6	63.0	90.0	33.5	2.12	NA
42	AML-M5	16.3	75.5	21.0	81.0	3.2	No
43	AML-M2a	104.8	75.0	43.0	114.0	0.70	No
44	AML-M0	119.6	83.5	85.0	124.0	0.84	No
45	AML-M1	19.4	98.0	15.0	91.0	1.51	Yes
46	AML	NA	NA	NA	NA	1.82	NA
47	AML-M2a	81.3	70.0	18.0	90.0	0.90	Yes
48	AML-M2a	39.0	65.5	50.0	93.0	1.65	No
49	AML-M5	17.7	56.0	134.0	63.0	0.36	No
50	AML-AML	244.0	NA	50.0	75.7	0.86	NA
51	AML-M5	65.2	91.0	74.0	100.0	0.89	No
52	AML-M2b	21.0	57.0	129.0	85.0	0.47	NA
53	AML	NA	NA	NA	NA	1.65	NA
54	AML-M2	22.2	29.0	20.0	105.0	1.18	Yes
55	AML-M2a	35.0	21.5	6.0	94.2	1.12	Yes
56	AML-M5	206.9	81.5	56.0	83.0	1.10	No
57	AML-M5	58.9	51.5	58.9	74.0	0.26	No

AML, acute myeloid leukemia; NA, not available; WBC, white blood cells; BM, bone marrow; PLT, platelet; Hb, hemoglobin.
